# Consumer Attitudes toward Pulses: Measuring the Implicit

**DOI:** 10.3390/nu15112608

**Published:** 2023-06-02

**Authors:** Juliana Melendrez-Ruiz, Gaëlle Arvisenet, Marie Dubot, Laurence Dujourdy, Stéphanie Chambaron

**Affiliations:** 1 Centre des Sciences du Goût et de l’Alimentation, Institut Agro Dijon, CNRS, INRAE, Université Bourgogne, F-21000 Dijon, France; gaelle.arvisenet@agrosupdijon.fr (G.A.); stephanie.chambaron-ginhac@inrae.fr (S.C.); 2 Terres Univia, 11 rue de Monceau, CEDEX 08, CS 60003, F-75378 Paris, France; m.dubot@terresunivia.fr; 3 Institut Agro Dijon—Service d’Appui à la Recherche, F-21000 Dijon, France; laurence.dujourdy@agrosupdijon.fr

**Keywords:** legumes, reaction time, sorting paired feature task, SPF

## Abstract

Research into consumer attitudes toward food products is important to help people adopt healthier, more sustainable diets. A positive attitude regarding an object is a prerequisite for its adoption. This study compares French consumers’ implicit attitudes toward pulses and cereals. Many studies have measured attitudes by explicit methodologies (e.g., questionnaires). Such methods are often biased by social desirability, and consumers may not be consciously aware of their attitudes toward food. A Sorting Paired Feature Task measures the strength of automatic associations, pairing images of pulses or cereals and adjectives with positive or negative valence. Participants sorted 120 paired stimuli as fast as possible. Pairs composed of pulses and negative adjectives were sorted faster than pairs composed of cereals and negative adjectives. Cereals with positive adjectives were sorted more rapidly than pulses with positive adjectives. Mistaken associations were more frequent for pairs composed of cereals and negative adjectives than for pairs composed of pulses and negative adjectives. These results highlight more negative implicit attitudes toward pulses than cereals. This study provides the first potential evidence of negative implicit attitudes toward pulses, which could explain the low consumption of these products.

## 1. Introduction

Consumers often find it difficult to adopt new eating habits, even when they are convinced of the need to adopt a more plant-based diet. Most of the studies about the barriers to the adoption of a more plant-based diet have focused on consumers’ unwillingness to reduce meat consumption. However, it is also crucial to ensure that consumers are able and ready to adopt new habits that will provide them with sufficient protein and a balanced intake of amino acids. Pulse consumption seems to be a key determinant of this dietary transition: the amino-acid pattern of pulses and cereals is complementary, and a diet containing both these food groups would satisfy adult dietary needs in amino acids while reducing the consumption of animal-based products [1]. Despite their numerous advantages for nutrition and sustainability, the consumption of beans, lentils, and chickpeas has decreased in France over the past hundred years [2,3]. Several barriers to pulse consumption have been proposed, including difficulty of preparation, dislike of the sensory properties of pulses, mental representations of pulses as products for vegetarians, and blurred categorization of pulse products in consumers’ minds [4,5,6,7]. This blurred categorization may result from the nutritional recommendation widely popularized in France between 2002 and 2017 by the National Plan for Nutrition and Health (PNNS), in which pulses were included, at the same level as cereals and tubers, in a rather broad category of starches: “Construct your main dishes around a serving of meat, fish, ham, or eggs, accompanied with vegetables and starches (potatoes, pasta, rice, wheat precooks, semolina, corn, pulses, etc.)” [8]. French consumers declared having “positive” attitudes toward pulses: respondents to a questionnaire considered these products to be good for health and eco-friendly [4], and participants in a free evocation task cited words that they then often evaluated as positive [7]. Infrequent substitution of meat by pulses has recently been attributed to the categorization of these products in different groups with very few common properties [9] or to the traditional main-dish structure, well anchored in French culture [5]. Our previous studies showed that French consumers, instead of using pulses as a substitute for meat, use them to replace cereals [5]. The FAO defines cereals as “annual plants yielding grains that belong to the grass family (Poaceae) and are cultivated for their edible seeds [10]. The cereals most commonly consumed in France are wheat, rice, corn, and oats [11]. Pulses are defined as leguminous crops harvested exclusively for dry seed (i.e., dried beans, lentils, and split peas), but excluding crops used mainly for oil extraction, e.g., soybeans, and those that are harvested green, e.g., green peas [12]. Both cereals and pulses provide carbohydrates and essential amino acids. Over past decades, pulse consumption has been considered strategic for dietary transition. Increasing consumption of pulses would guarantee a satisfying protein intake when decreasing meat consumption. In order to provide all the essential amino acids, the daily diet should contain both cereals and pulses. However, the figures observed in 2020 for consumption in France were 126 kg/person for cereals, and only 2.5 kg/person for pulses [13]. For French consumers, pulses and cereals are considered as equivalent in the main-dish structure and used as accompaniment for meat, which is considered to be the central element of the traditional French meal. It is perhaps surprising that the figures for these two food groups are so different even though cereals and pulses are considered equivalent. Consumer perceptions, whether positive or negative, shape attitudes toward pulses, which then shape consumption behavior together with personal sensory preferences, health concerns, and cultural background. The methods used in these studies seem to have preferentially elicited explicit attitudes. This study was therefore specifically designed to measure implicit attitudes toward pulses.

Attitudes toward a product are usually formed by previous experience with this product or a similar product from the same category [14]. Even without previous experience with a product [15], attitudes can be shaped from what a person has seen or heard from others or from advertising. Therefore, attitudes are thought to influence food choices and allow the evaluation of a specific object to be favorable or unfavorable [15], with varying degrees of favor or disfavor [16]. If consumers feel favorable toward a product, attitude can facilitate its choice and/or purchase. By contrast, a negative attitude toward an object or concept can hinder its use or adoption. Attitudes can be measured using explicit or implicit methodologies. Methods used to obtain explicit measures (e.g., self-administered questionnaires) present several drawbacks. Participants’ answers may be strongly influenced by many external factors (e.g., social desirability, difficulty of information retrieval) [17]. To avoid these drawbacks, implicit methods do not focus on participants’ answers but on their automatic reactions or physiological responses [18]. Some tests capture the psychological aspects related to cognition, judgment, and actions, which may not be described by participants when questioned directly, or which are not accessible by examining personal thoughts and feelings [19]. The implicit cognition process is not conscious but automatic, unrelated to judgment or social behavior [20]. Successful measurement of implicit attitudes requires an automatic outcome, only recorded when participants remain ignorant of the true research aim. A study in which some participants became aware of the presence of non-attentively perceived olfactory cues and consequently changed their behavior provides tangible evidence of how automatic processes can be impacted [21].

Some implicit tasks rely on the measurement of reaction time to access mental processes that cannot easily be assessed by direct or verbal measures. The most famous of these tasks is the Implicit Association Test (IAT), which measures the strength of automatic associations between concepts [22]. However, the IAT suffers from many drawbacks, and other tests have been developed to overcome its limits. The Sorting Paired Feature Task (SPF) is an example of these alternative tests, allowing researchers to assess the strength of sub-associations [23]. In the SPF, two stimuli are presented at the same time, which makes this test more sensitive than the IAT measures to stimulus meanings [24].

The design of an SPF task requests two categories of objects to be compared. These two categories must belong to the same broader category. For this reason, in the present study, cereals were chosen as the alternative product group to be compared to pulses. The objective of the present study was to measures the implicit attitudes of French non-vegetarian participants toward pulses and cereals by adapting the SPF task. Our hypothesis was that participants would have more negative implicit attitudes toward pulses than toward cereals.

## 2. Materials and Methods

### 2.1. Participants

To qualify for the study, participants (*n* = 95) had to be aged over 18 and under 65 (Table 1), without specific or restrictive diets (e.g., vegetarian, vegan, etc.). To be able to participate in the study, participants had to have access to the Internet and a computer with an AZERTY keyboard. The participants were informed about the conditions of the study and were remunerated at the end with a 10-euro voucher. The sample size required to reach a power of 0.9 with an alpha of 0.05 was 54 participants; the power with 95 participants was 0.993. The participants completed the task once, from home, using a link that was sent to them. The participants were recruited in the area of Dijon (France), from the Chemosens Platform’s PanelSens database. This database complies with national data protection guidelines and has been examined by French National authorities (Commission Nationale Informatique et Libertés—CNIL—135n = 1.148.039). The study was conducted in accordance with the Declaration of Helsinki and was approved by the local ethical committee of INSERM N° 21-873 (Institutional Review Board INSERM or CEEI, IRB00003888, IORG0003254, FWA00005831).

### 2.2. Construction of the SPF Task

#### 2.2.1. Selection of the Stimuli

**Figure 1:**
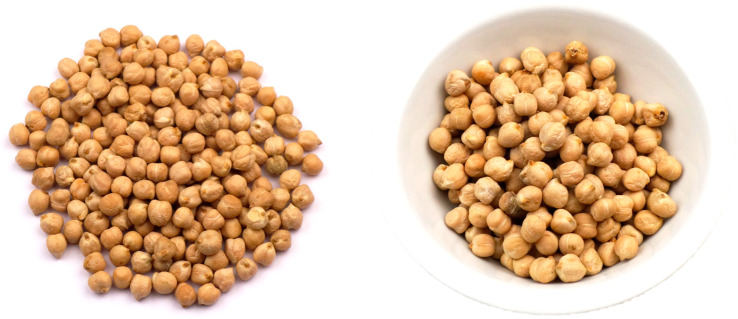
Example of the two images for chickpeas.

All stimuli were composed of a word and an image. Words (*n* = 12) were used to represent attitude attributes (6 good and 6 bad valence); images (*n* = 16) were used to represent the two object categories, pulses and cereals (8 stimuli each). The positive attitude attributes were good, attractive, tasty, delicious, interesting, and pleasing. The negative attitudes were bad, repulsive, disgusting, insipid, indifferent, and boring. To obtain the images, four products were photographed in two different presentations with a white background. The angle and the light were maintained constant for all products (Figure 1). For pulses, uncooked green lentils, red beans, split peas, and chickpeas were selected; for cereals, uncooked long-grain rice, curved macaroni, semolina, and corn were selected.

#### 2.2.2. Structure and Measures

The four paired categories (pulses + positive; pulses + negative; cereals + positive; cereals + negative) were presented in the four corners of the screen [24,25]. Each corner corresponded to a response key: top left = A, bottom left = E, top right = I and bottom right = P. As in the original SPF task, the positive attitude attributes were always shown on the left side; the negative attitude attributes were always shown on the right side [24], position of the object category (pulse and cereal) was randomized between participants.

**Figure 2:**
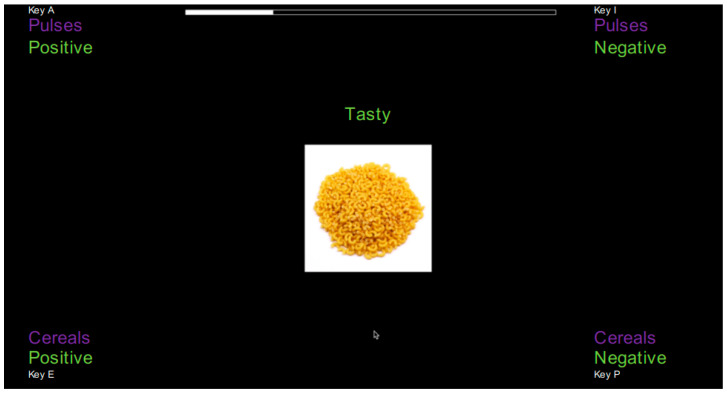
Presentation of the screen to participants (the original text was in French).

At set intervals, a word and a food product image appeared at the center of the screen. The word was always above the image, as shown in Figure 2. The task consisted of a total of 3 blocks with 40 trials each (10 trials for each pair of categories). The order of the three blocks was randomized between participants. For the implicit measures, the inter-trial interval was set at 300 ms [24]. Before the start of the task, the participants were given a quick training block with two animal images (bird and eagle) with positive and negative words.

The participants were instructed to classify each pair of categories as quickly as possible in one of the four paired categories by touching one of the keys. If a wrong answer was given, a red cross appeared on the screen, and participants had to correct their response. As shown in Figure 2, presenting the word “tasty” and an image of pasta, the correct response would be to classify these two stimuli in the corner labelled Positive + Cereals by pressing the response key E.

### 2.3. Statistical Analysis

#### 2.3.1. Data Processing

The data obtained from the SPF task rely on response latency (reaction time—RT, in milliseconds) and the number of errors. The training block was excluded as well as all the trials with the lower tail deletion (<400 ms) and upper tail deletion (>10,000 ms) as suggested for the original SPF task [26].

#### 2.3.2. Data Analysis

A two-way permutational ANOVA was performed over a table containing attributes (good and bad), object categories (pulses and cereals), and reaction time (RT) in columns, with participants in lines. If significant differences were found, the ANOVA analyses were followed by a Tukey’s HSD test for multiple comparisons between and within groups [27,28].

Descriptive data analysis was obtained by calculating the mean values for all the RTs obtained and the sum of the errors for participants.

The frequency of errors made by a participant for each paired category (pulses + positive; pulses + negative; cereals + positive; cereals + negative) was calculated and then converted into a rank. The paired category for which a participant made more frequent errors obtained the highest rank. A Friedman test on the sum of ranks was used to determine if there were significant differences among the paired categories. A multiple pairwise comparison using the Nemenyi/two-tailed test was then used to compare the frequency of errors made in each paired category [29].

Data analyses were carried out using RStudio 2023.03.0 and R-4.2.1 with specific packages: lmPerm, permuco [28], agricolae [30], and PMCMRplus [31]. The lmPerm and permuco packages were used to compute permutational ANOVA. Agricolae and PMCMplus were used to compute Friedman and Nemenyi tests.

## 3. Results

### 3.1. Reaction Time for the Categorization of Paired Images and Attributes

The permutational ANOVA allowed us to compare the effect on reaction time (RT) of attitude attributes (good and bad) and object categories (pulses and cereals).

**Figure 3:**
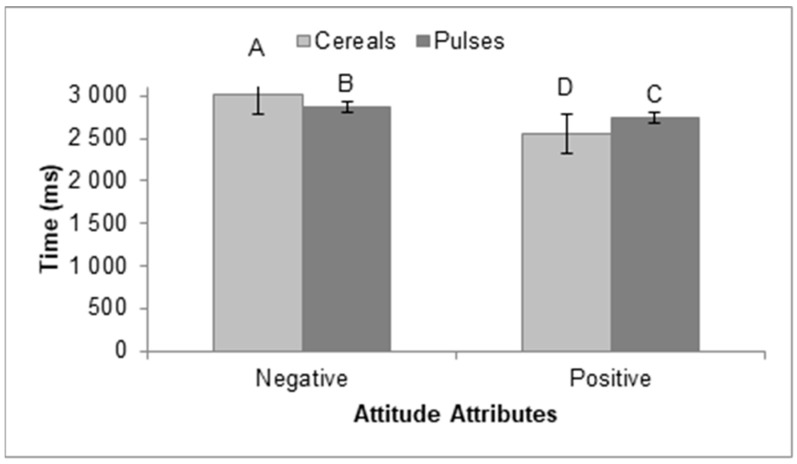
Mean reaction time (RT) spent to categorize positive and negative attributes for each object category. Paired categories sharing the same letter are not significantly different (Tukey’s HSD test; *p* < 0.05).

Between groups, there was no significant difference in RT for object or attitude at the significance level of 0.05. Within groups, however, there was a significant difference in RT for object (F (1) = [2.853], *p* = [<0.000]) and for attitude (F (1) = [2.477], *p* = [<0.000]), and there was a significant interaction between both variables (F (1) = [7.944], *p* = [<0.000]). A Tukey’s HSD test for multiple comparisons was performed on this interaction. The results presented in Figure 3 show that participants categorized negative adjectives faster when they were associated with an image of pulses than when they were presented with an image of cereals. By contrast, participants categorized positive adjectives faster when they were associated with cereals than when they were associated with pulses (Figure 3).

### 3.2. Descriptive Results

The total mean reaction time (in ms) taken by participants was 2.94 s for all products paired with a negative adjective, with a total of 1519 errors. When paired with positive adjectives, the mean time was 2.64 s, with 1388 errors (Table 2).

**Figure 4:**
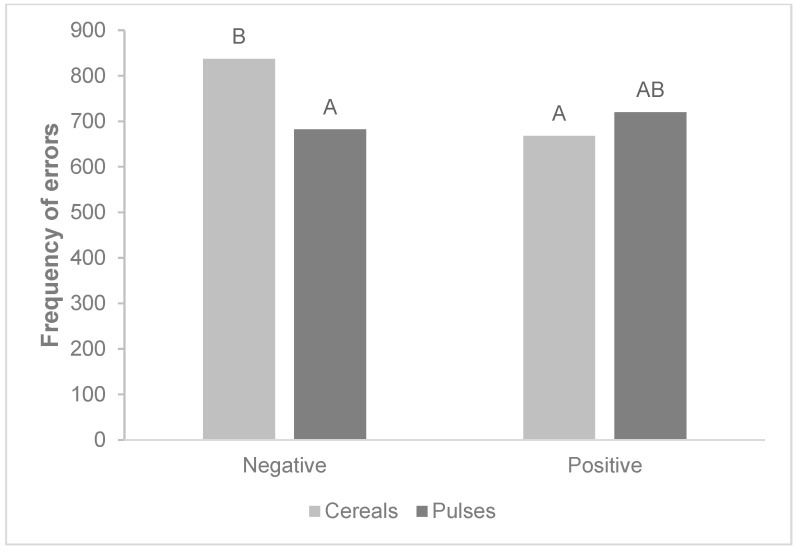
Total frequency of errors made by participants during the SPF task. Paired categories sharing the same letter are not significantly different (Nemenyi/two-tailed test; *p* < 0.05).

The Friedman test showed significant differences regarding the total number of errors (*p*-value < 0.0001). Participants made significantly fewer errors when categorizing negative adjectives with pulses than when categorizing them with cereals. Errors in categorization of positive adjectives occurred at a similar frequency whether they were associated with pulses or cereals (Figure 4).

## 4. Discussion

### 4.1. Dualism between Implicit and Explicit Attitudes toward Pulses

The objective of the present study was to measure the implicit attitudes of French non-vegetarian participants toward pulses and cereals by using an implicit methodology: the SPF task. It was hypothesized that participants would have more negative implicit attitudes toward pulses than toward cereals.

More negative implicit attitudes were indeed found toward pulses than toward cereals, since the participants required less time and made fewer mistakes when categorizing pulses paired with negative adjectives. These results confirmed our hypothesis. Reaction times are assumed to reflect the underlying computations required for decision making and action planning. A faster reaction time reveals that items are easily computed by participants, as they depend on automated processes, while difficult-to-compute items require more controlled processing, which increases the time spent on the task [32]. Another reason that could explain why participants in our study took more time to associate pulses with positive adjectives and cereals with negative adjectives is a possible decision conflict [33]. A stimulus that generates higher conflict slows down participants’ cognitive processes and therefore slows down their reaction time [34]. A cognitive internal conflict may occur when cereals are associated with negative adjectives because, in the minds of participants, such products elicit positive attitudes. Similarly, the association of pulses with positive adjectives could create a conflict, as pulses elicit more negative attitudes. Reaction time and number of errors were used as attentional performance measures, i.e., longer reaction times and higher error rates were associated with poorer attentional performance. The number of errors is a critical element in the cognitive framework. Reaction time may be linked to a certain level of decision uncertainty: slow responses could be related to decreased attention that delays RT [35]. In our results, reaction time and number of errors reflected not only cognitive processes (controlled or automatic), but also the content of the mental representation related to the object (pulses and cereals in this study).

In studies using questionnaires, i.e., a direct task, the instruction given and the time allowed to answer the questions is long enough for participants to voluntarily and consciously retrieve information stored in memory [36], and thus explicit attitudes can be measured. By contrast, in the present study, the instructions given and the experimental design (short presentation time for the stimuli) do not allow controlled decision making; thus, attitudes measured in these conditions can only be implicit. These differences in study design may explain the discrepancies between our results and those presented in the literature: French participants have “positive” attitudes toward pulses when they are explicitly questioned, but they have “negative” attitudes when they are indirectly questioned. Conflicting results obtained with implicit and explicit tasks may be due to differences in the nature, sensitivity, motivation, and cognitive biases measured by these tasks. Implicit and explicit tasks measure different forms of information processing. Explicit tasks measure a person’s ability to consciously report thoughts, feelings, and behaviors, while implicit tasks measure a person’s ability to process information without being aware of their thoughts, feelings, and behaviors.

The dual attitudes model could explain the discrepancies between the results obtained with direct and implicit tasks [37]. This model postulates that when an individual has the motivation and the opportunity to think carefully about a decision, behavior will be driven by deliberate processes. By contrast, if an individual does not have the time or cognitive resources to think about a decision, behavior will be driven by spontaneous processes. This model considers that different evaluations (implicit and explicit) of the same object can coexist simultaneously, and both can influence behavior [37]. According to the authors Brannon and Gawronski, “old attitudes may not be erased from memory when people change their attitudes in response to new information” [38]. In this sense, one person may have both attitudes toward pulses: a negative implicit attitude that could reflect their avoidance of pulses, and a positive explicit attitude that could be related to exposure to positive information about pulses (nutrition, health, environment), or positive consequences of pulse consumption (satiation, appreciation of organoleptic properties). Consequently, these inconsistencies between implicit and explicit attitudes related to pulses could be explained by multiple representations inserted in separate mental networks, inducing contradictory beliefs [39]. Another theoretical model could also explain these differences between explicit and implicit attitudes: the Reflective–Impulsive Model [40] (RIM) of behavior regulation proposed by Strack and Deutsch (2004). According to the RIM, two mental systems jointly influence behavior in response to the environment. In doing so, the two systems follow different principles and operate under different conditions [40,41].

Implicit attitudes about food are formed gradually and are malleable; they are strengthened and adjusted over time in response to repeated consumption of a food item [42], which will increase familiarity and routine behavior [43]. A study found that implicit attitudes about natural food were better explained by repeated exposure over a long period [34]. Even though the product is not the same as in our study, such a result could explain why our participants had more positive implicit attitudes toward cereal than toward pulses. Cereals are considered a staple food in France and have been part of French food habits for a long time, while pulses have occupied an ever more limited place in consumer dishes over the past few decades [2,44]. Interest in pulses has increased very recently, with the need to find new sources of protein. In the context of a dietary transition, where pulses are particularly valorized by public health agencies, an evolution of explicit attitudes toward this product category may be observed in the years to come. But will implicit attitudes toward pulses change?

The challenge is to change implicit attitudes, and a recent systematic review highlighted the “predictive validity of implicit measures on the actual food behavior” [45]. Having a positive attitude toward pulses is a prerequisite to increasing their consumption and improving the observance of dietary recommendations.

### 4.2. Perspectives: Can Consumer Behavior Be Changed despite Implicit Negative Attitudes?

Encouraging new attitudes, change, or adoption of new behaviors in consumers is difficult. The construction of attitudes comes from the interaction of the environment (social, cultural, and physical) with the individual’s psychological and physiological characteristics [14]. Although attitudes are seen as a result of prior experiences, they can change over time, and are incremented after storage in memory under the form of a network of associative knowledge [46]. Consequently, to overturn implicit attitudes toward an object, it is necessary to tackle previously existing representations, which is considered a rather difficult task [39]. One study found that exposing participants to the repeated pairing of unhealthy food (snacks) with negative stimuli and healthy food (fruits) with positive stimuli can shift food evaluation assessed by implicit measurement, even if no change in behavior was observed [47]. Several psychological theories and techniques could help change implicit attitudes.

The first way to change implicit attitudes is by means of evaluative conditioning (EC), which consists in forming an attitude toward an object or changing its formation by pairing the object with positive or negative stimuli [48]. The positive or negative valence of the stimuli is progressively “transferred” to the attitude toward the object. Another way to change implicit attitudes is based on the Cognitive Dissonance Theory: this theory suggests that individuals experience discomfort when their attitudes and behaviors are inconsistent. To reduce this discomfort, individuals may adjust their attitudes to align with their behavior. Marketers can leverage this phenomenon by creating advertisements or campaigns that encourage consumers to act in ways that are consistent with the desired implicit attitudes. For example, a campaign that encourages recycling may lead to more positive implicit attitudes toward the environment. The influence of peers can also be determinant. The Social Learning Theory suggests that individuals learn attitudes and behaviors by observing others [49]. Featuring customer testimonials or endorsements from influential people may lead to more positive implicit attitudes toward the object of interest [50]. In the case of pulses, famous chefs or culinary influencers who use pulses and communicate about their interest in these products on social networks could serve as “social influencers”, and individuals who follow them could develop more positive implicit attitudes toward pulses and progressively include them in their menus.

Given the difficulty of changing implicit attitudes, other strategies could be implemented to impact consumer behavior. Nudging could represent a suitable strategy. “Nudging” means “subtly guiding”. According to Thaler and Sunstein, a nudge is “any aspect of the choice architecture that alters people’s behavior predictably without forbidding any options or significantly changing their economic incentives” [51]. The choice architecture refers to the idea that changes in the environment where a decision is made can affect individual decision making, and thus behavior, while always preserving the freedom of choice [52]. A nudge strategy is characterized by four central elements (i) it does not rely on explicitly indicating “what to do” to consumers, (ii) nudges should always encourage a more socially desirable choice, by favoring healthier or more sustainable behaviors, or decreasing the negative ones, (iii) nudges should not remove choice options, and finally, (iv) nudge strategies are usually small, easy to implement, and inexpensive [53]. The design of a nudging study focuses on reshaping the choice architecture to obtain from consumers a desirable choice or behavior, whatever their implicit attitudes [54].

This contribution is a first use of an implicit task in the food domain to try to explain the observed divergence between the recommended diet and consumer food habits. Indeed, knowing consumers’ implicit attitudes could allow researchers to better understand the discrepancy between what consumers declare and their actual consumption of pulses, which remains low. One limit of our study is that participants performed the task at home and that it was not possible to control the participants’ attention span. Thus, some attentional lapses may have occurred during the task [35]. By eliminating all the measures above and below the threshold proposed by previous studies, this risk was lessened. Another point of concern is the choice of stimuli, which is crucial to the results obtained by the SPF. Given that this study is a first in the food domain, it would be interesting to adapt it with the use of different stimuli borrowed from other food categories. In addition, given that culture is a determinant of dietary behavior, it would be interesting to replicate this study in another country where pulse consumption is more embedded in food habits.

## 5. Conclusions

Changing implicit attitudes can be challenging, as they are deeply ingrained and often non-conscious. Switching implicit negative attitudes toward pulses in French consumers will require increased familiarity with these products. To achieve this goal, it would be necessary, as a first step, to provide more information about what pulses are, their long-term benefits, and their unique characteristics. It would also be necessary to create highly persuasive messages and information to associate pulses with a positive valence. It could be interesting to create interventions that combine both implicit training and explicit information, which could have a positive impact; the newly acquired positive attitudes could thus translate into a change of intention and behavior.

## Figures and Tables

**Table 1:** Main demographic characteristics of the participants.

Characteristics	Categories	Number of Participants	Porcentage
Sex	Male	36	38%
	Female	59	62%
Age	18–24 years	7	8%
	25–34 years	28	29%
	35–44 years	16	17%
	45–54 years	27	28%
	55–65 years	17	18%
Education *	High	47	49%
	Medium	39	41%
	Low	9	10%

* Level of education expressed in number of years of formal schooling: Low, <12; Medium, ≥12 and <15; High, ≥15.

**Table 2:** Mean reaction time (in ms), number of errors, and percentage of errors made by participants during the SPF task for each category.

	Negative				Positive			
Product	Mean (ms)	(SD)	Errors	%	Mean (ms)	(SD)	Errors	%
Cereals	3006.783	1909.2	837	55	2543.194	1713.0	668	48
Pulses	2870.934	1942.1	682	45	2741.291	1790.5	720	52

## Data Availability

Data are available upon request.
